# Psychosocial Resources for Hedonic Balance, Life Satisfaction and Happiness in the Elderly: A Path Analysis

**DOI:** 10.3390/ijerph17165684

**Published:** 2020-08-06

**Authors:** Raquel Lara, Mᵃ Luisa Vázquez, Adelaida Ogallar, Débora Godoy-Izquierdo

**Affiliations:** 1 Grupo de Investigación Psicología de la Salud y Medicina Conductual (CTS-267), Facultad de Psicología, Universidad de Granada, Campus Universitario de Cartuja, 18071 Granada, Spain; rlaramoreno@ugr.es (R.L.); mlvazquez@fundacionsafa.es (M.L.V.); adelaidaogallar@ugr.es (A.O.); 2 Departamento de Psicología Social, Facultad de Psicología, Universidad de Granada, Campus Universitario de Cartuja, 18071 Granada, Spain; 3 Departamento de Psicología Evolutiva y de la Educación, Centro Universitario Sagrada Familia, Úbeda, 23400 Jaén, Spain; 4 Departamento de Personalidad, Evaluación y Tratamiento Psicológico, Facultad de Psicología, Universidad de Granada, Campus Universitario de Cartuja, 18071 Granada, Spain

**Keywords:** happiness, subjective well-being, psychosocial resources, older adults, path analysis

## Abstract

We explored possible paths from physical and mental health-related quality of life, self-efficacy, optimism, and social support to happiness in older adults, considering hedonic balance and life satisfaction as mediators. A total of 154 Spanish male and female (50%) older adults (65–96 years old, M = 77.44, SD = 8.03; 64% noninstitutionalized elderly) voluntarily participated in this correlational, cross-sectional study. The participants completed self-reports on their perceived health status, self-efficacy, social support, optimism, and global subjective well-being (SWB) as well as its dimensions. Path analysis was used to examine direct and indirect relationships. The final model had an excellent fit with the data (χ2(10) = 11.837, *p* = 0.296, χ2/df = 1.184; SRMR = 0.050, CFI = 0.994, RMSEA = 0.035), revealing the unique causal effects of all the included predictors on happiness. With the exception of self-efficacy, the psychosocial resources predicted older adults’ current happiness, and this relationship was fully mediated by hedonic balance and life satisfaction, which were found to be putative intermediary factors for SWB. Self-efficacy in turn predicted the remaining psychosocial resources. Our findings extend the existing evidence on the influences of health-related quality of life, self-efficacy, optimism, and social support on SWB. Furthermore, they support the proposal of hedonic balance and life satisfaction as dimensions of SWB, thus supporting the tripartite hierarchical model of happiness. These results may inform future interventions seeking to improve happiness in late adulthood.

## 1. Introduction

Research on the well-being of older adults has traditionally focused on negative emotions and psychological distress rather than the value of positive states [1]. This trend is changing, as researchers have begun to investigate the adaptive function of positive emotions and personal satisfaction and their impact on elderly people’s happiness [2]. In the last two decades, there has been growing interest in how personal resources and psychosocial factors promote adaptation and quality of life and contribute to the well-being of older adults [3]. As life expectancy increases worldwide, the need for scientific work on the positive correlates of health and well-being in later life stages increases to ensure that longevity is accompanied by sustained subjective well-being (SWB), even in the presence of major life events, declines and changes in living circumstances. Thus, the paradigm of “successful aging” has shifted to include SWB to promote aging well in later life stages in public health policies [1,2,3,4]. In addition, interventions focused on enhancing SWB have been shown to be powerful applied resources for transforming the ordinary lives of many people into lives that are fully satisfying and meaningful [5,6], also in older adults [7,8,9].

As proposed in Diener’s tripartite model of SWB [10,11,12], affect or hedonic balance (HB) is one of the two components of happiness, in addition to life satisfaction (LS). HB, the affective component of SWB, refers to the emotional responses occurring when we make judgments about our life, which can be either positive (e.g., joy, pleasure, euphoria) or negative (e.g., fear, anger, sadness) [13]. A positive HB reflects the experimentation of positive emotions at a higher level than negative affect, whereas negative HB results from the contrary. Evidence suggests that happiness is most closely tied to the frequency and duration of emotional experiences, instead of their intensity [14]. LS, the cognitive component of SWB, reflects an individual’s judgment about the compatibility of their own living circumstances with personally established standards [15]. LS judgments are grounded on personal experiences with the work, health, family, and social domains of life and are referred to as (positive) evaluations of overall quality of life as a whole [11,16]. As a consequence, a person is (self) considered to be happy when he or she has high levels of both HB and LS [11,17].

### Associations between Affect Balance, Life Satisfaction and Happiness with Psychosocial Variables in Older Adults

A number of studies have found that as people grow old, their HB and LS decrease [18,19,20,21,22,23]. In addition, in general, evidence supports that higher levels of SWB (i.e., HB and LS) are linked with more positive indicators of psychological, physical, interpersonal, and socioeconomic functioning at both the personal and societal levels [17]. Moreover, abundant research has shown that both HB and LS are associated with different psychosocial resources in older adults, including self-efficacy, optimism, social support, physical and mental health, and quality of life, which might explain the age-happiness relationship, as we review below. The reduced positive affect and LS and the augmented experimentation of negative emotions observed in late adulthood can be due to not only the increased likelihood of experiencing negative events in this life stage (e.g., the death of relatives or friends, physical and mental health issues, stress, loneliness, economic difficulties, etc.) [21] but also a lack of psychosocial resources for feeling happy [24].

For example, positive social resources (e.g., social support, relationships with family and friends, interpersonal activities, etc.) are correlated with positive affect and LS and inversely correlated with negative affect in older adult populations [25,26,27,28,29,30,31,32,33,34]. Different facets of social support differentially predict the components of SWB. For instance, the number of social resources or activities is not as important as their quality; thus, loneliness is experienced with lower negative affect among people integrated in the context of social relationships of high quality [35,36,37]. In addition, both providing and receiving social support have an effect on positive affect and LS, while receiving social support is also associated with ill-being indicators such as negative affect and depression [29,31,34]. Systematic reviews [38,39] have noted that the overall relationships between social support and HB, LS, and SWB are positive. In conclusion, evidence suggests that social relationships improve older people’s well-being, leading them to rate their lives as more fulfilling and satisfactory and to experience more positive and less negative affective states.

Aging is often associated with several health-related negative conditions (e.g., depression, poor daily functioning, cognitive and physical deterioration, chronic illnesses). Elderly people generally report having a more positive HB and higher LS when they demonstrate having better mental and physical health as well as a better functional status and greater ability to perform daily activities [27,29,32,33,39,40,41,42,43,44,45]. It has been proposed that mental health can be even more important than physical health and functionality [30]. In addition, an active lifestyle has also been associated with a better health status, positive affect, higher LS, and greater happiness [21,42,46]. Poor self-perceived health in older adults is associated with higher negative affect and lower LS, and these effects can be increased in older adults who have little perceived control/self-efficacy and high levels of frailty [40,47].

Perceptions of controllability and self-efficacy are important for successfully and satisfactorily managing aging [48,49]. High levels of self-efficacy, control, mastery, autonomy, and coping resources empower older adults to better manage aging, thereby enhancing their well-being, LS, and HB [26,44,50,51,52,53]. In contrast, low levels of self-efficacy are associated with poorer mental health and daily functioning as well as lower levels of well-being and LS.

Optimism has also been commonly associated with positive affect, LS, and happiness among older adults [44,45,52]. However, other researchers have found that its effect is partially mediated by other variables, such as social support [54] or participation in daily social activities [55].

In short, it can be affirmed that successful and happy older adults are those who have good physical and mental health and greater health-related quality of life, as well as high levels of optimism, social support, and self-efficacy. Nevertheless, although many of the abovementioned studies used modeling (e.g., structural equation, path analysis) for analysis, none of them investigated the contribution of all these psychosocial resources to HB, LS, and thus SWB.

The aim of the present study was to create an explanatory model of all these relationships by establishing the direct and indirect (i.e., mediating) paths among these psychosocial variables; optimism, self-efficacy, social support as well as health-related quality of life were considered as potential predictors, HB and LS as mediating variables, and happiness as the main outcome. To our knowledge, no previous studies have investigated the combination of all the proposed resources and outcomes in people 65 years old and older. In addition, we assessed SWB with a global happiness indicator, and positive and negative affect (i.e., HB) and LS as its contributors, on the basis of the tripartite model of SWB [10] and the suggestions made by other researchers [56], allowing the modeling of the shared variance among the components as reflected in the latent SWB factor as well as the unique variances of each component, independent of the latent factor. Since HB and LS are different but related constructs and may have different causes and correlates [57], we decided to include both factors in the analyses to examine the specific interplay between the predictor psychosocial variables and these dimensions and, thus, happiness.

## 2. Materials and Methods

### 2.1. Participants

**Figure 1:**
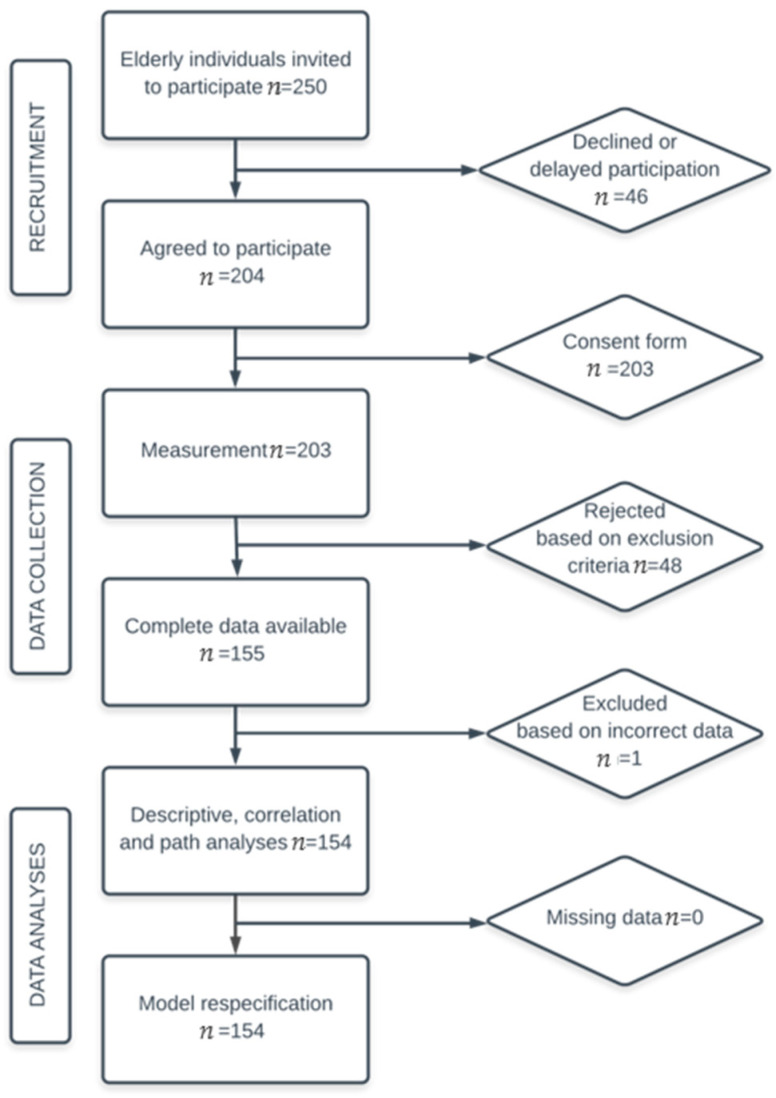
Study flowchart.

After informed consent was provided, 154 Spanish men and women aged 65 to 96 years voluntarily participated in this study. The participants were recruited from private homes, public places (e.g., parks, churches, health centers), neighborhood associations, nursing and geriatric homes, day centers, recreational centers for adults, and schools for adults in a province in southern Spain by convenience sampling. The older adults were invited to participate in a larger study on quality of life during late adulthood. For the purposes of the present study, only individuals—particularly those who were institutionalized—without a diagnosis of depression, dementia, or any other severe neurological or psychopathological disorder were selected from a larger population of older adults initially recruited, which together with an age below 65 years and above 100 years were the only exclusion criteria. Figure 1 shows the study flowchart.

Sample size was over the estimated sample size of 84 participants for alpha = 0.05, beta = 0.2 (power = 0.8), correlation coefficient *r*_0_ = 0 and estimated correlation coefficient *r*_1_ = 0.3 for correlational analyses [58,59]. The participants in this study can be considered comparable to the general older population in southern Spain in terms of the sociodemographic conditions, according to national databases, such as the National Statistics Institute, Women Institute and Elders and Social Services Institute (IMSERSO) [20,24].

### 2.2. Measures

The Happiness Scale (HS) [20] assesses current happiness (i.e., in the last few days or weeks) and general or past happiness (during one’s lifetime) using two questions (“How happy are you in the last few days or weeks/were you during your lifetime?”), to which participants responded with a score on a scale from 0 = extremely unhappy to 10 = extremely happy. The HS also includes a list of 20 life dimensions (e.g., family and family relationships, work, leisure time and hobbies) for which the respondent must indicate the degree to which he/she perceives that each one contributes to his/her current happiness (0 = not at all, 10 = very much). Only the current happiness score was used in this study. This self-report has been previously used in research conducted in older adults [19,20,24].

The Affect Balance Scale (ABS) [60], Spanish version [61], was used to assess positive affect (9 items) and negative affect (9 items). The partial scores were obtained by summing the responses (1 = not at all, 3 = completely), and the total affect balance score was obtained by subtracting the negative affect score from the positive affect score, with a positive score indicating a predominance of positive affect. The ABS assesses the frequency of emotional experiences during the last week, thus allowing the frequency vs. the intensity of affective states to be evaluated in accordance with Diener’s proposal [10]. The psychometric properties of the ABS have been well established in the Spanish population [61,62].

The Life Satisfaction Scale (LSS) [15,63], Spanish version [64], was used to assess the participants’ general levels of satisfaction with their lives, and it includes 5 items (1 = completely disagree, 7 = completely agree). Higher scores indicate greater overall satisfaction with life. The psychometric properties of the LSS have been widely established in the Spanish adult population [62,64].

Health-Related Quality of Life was measured using the Medical Outcomes Study Short Form-12 (SF-12, version I) [65], Spanish version [66], which contains 12 self-reported items pertaining to physical and mental health functioning. Separate norm-based composite scores ranging from 0 (poor) to 100 (excellent) were computed; the mean score is 50, and the standard deviation is 10. Higher scores on the SF-12 scale indicate better physical and mental functioning and well-being. For the present study, we used the scores of the physical component summary (PCS) and the mental component summary (MCS), which were calculated based on specific norms for the Spanish population [67]. The SF-12 is a reliable and valid measure commonly used in population surveys. Its psychometric properties have also been shown in Spanish study populations [67,68,69].

The General Self-Efficacy Scale (SES) [70], Spanish version [20], was used to assess self-efficacy perceptions in two main areas, general self-efficacy and social self-efficacy, and it includes 30 items (1 = strongly disagree, 5 = strongly agree). The total score was calculated by summing the scores for both dimensions. Higher scores indicate greater perceived personal efficacy. This scale’s psychometric properties have been established in a Spanish elderly population [71]. In this study, control items were removed to shorten the assessment protocol.

The Life Orientation Test, revised version (LOT-R) [72], Spanish version [73], was used to assess optimism; it has 10 items (1 = strongly disagree, 5 = strongly agree), with higher scores indicating higher optimistic expectancies. The psychometric properties of the LOT have been widely established in a Spanish population [73]. In this study, control items were removed to shorten the assessment protocol.

The MOS Social Support Scale (MOSS) [74], Spanish version [75], was used to assess social support, including the emotional/informational, tangible, affectionate, and positive social interactions dimensions with 20 items (1 = never, 5 = always); the scores indicate the frequency with which each type of available support was given. The global perceived social support score was obtained, with higher scores indicating greater perceived social support. The number of available relatives and friends was also reported, but this information was not considered for analysis. The psychometric properties of the MOSS have been well established in a Spanish population [76].

Participants also reported their age, sex/gender, marital status, education level, employment status, monthly family income, and number of children. They also indicated their perceived health status on a face-validity question (0 = very poor, 10 = excellent); whether they were suffering from any disease at the time of the study and if so, their diagnosis/diagnoses; and whether they were undergoing any kind of treatment or therapy and if so, the type(s).

### 2.3. Study Design, Procedures, and Analytical Plan

This is a correlational, cross-sectional study. We provided all potential participants with general information regarding the main objective of the study to request their voluntary participation. Next, after the individuals who decided to participate were provided with detailed information on the study and their tasks and rights as a participant, they signed a written consent form, and they then received specific instructions on how to complete the questionnaires correctly. The assessment tools were counterbalanced to avoid order bias. Applying was collective, but due to personal characteristics or circumstances, assessment was individualized or, in some cases, administered in an interview format (e.g., severe vision deterioration, motor difficulties). All participants completed the questionnaires during a single session. This study was approved by the Ethic Committee for Human Research of the University of Granada in June, 2011; recruitment, data gathering, and initial analyses were conducted between June and September, 2011.

Firstly, preliminary and exploratory data analyses were conducted to detect (and correct) possible errors in the data and missing data as well as to select the statistical tests to be used. A few variables were not normally distributed (Shapiro-Wilk test *p* < 0.05); however, Levene’s tests confirmed homoscedasticity for all variables (*p* > 0.05). Univariate and multivariate outliers were identified before the analyses were conducted. Potential outliers, based on the distribution of Z scores and Mahalanobis’ test results, were considered unusual but possible values, and thus, no participant was excluded from the analyses. No multicollinearity among the independent variables was detected based on the variance inflation factor (VIF), the tolerance parameter and the condition index. Consequently, none of the variables were excluded from or combined for the analyses. 

**Figure 2:**
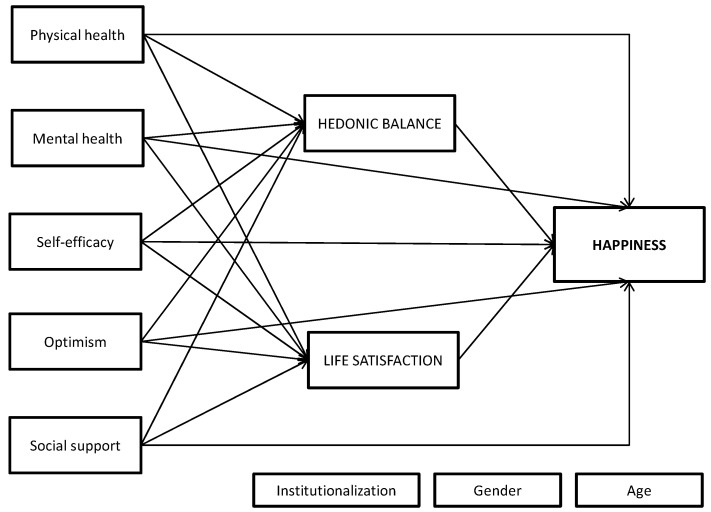
Hypothesized model. Observed, endogenous variables: Hedonic balance, life satisfaction and happiness. Observed, exogenous variables: Physical health, mental health, self-efficacy, optimism and social support. Controlled confounders, exogenous fixed variables: Institutionalization status, age and sex/gender.

**Figure 3:**
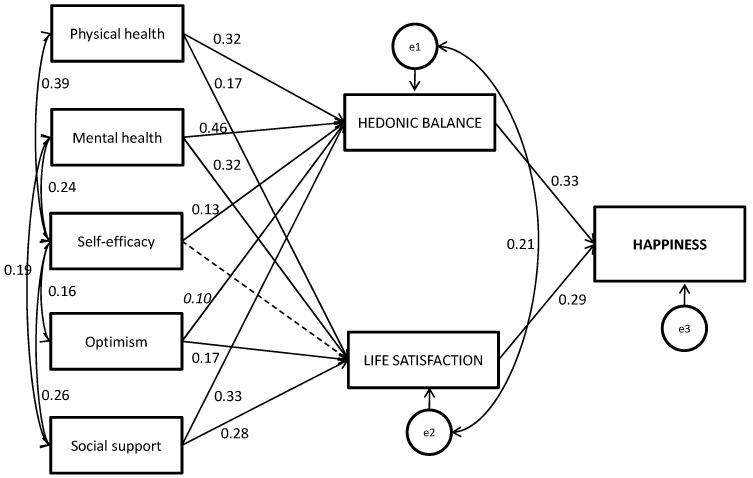
Path model explaining the causal relationship from physical and mental perceived health, self-efficacy, optimism, and social support to happiness with hedonic balance and life satisfaction as mediating variables. Standardized path coefficients are presented. Data from *continuous arrows* represent significant standardized regression weights (parameter estimates) (two-tailed *p*-values, *p* < 0.05), data in italics are non-significant at *p* < 0.10, and data from *dotted arrows* represent non-significant standardized regression weights. e_i_: error terms for each variable. *N* = 154. Bootstrapping: 1000 samples, 95% bias-corrected confidence interval.

Thus, in addition to descriptive analyses, parametric correlation analyses were conducted. Then, to explore the relationships between mental and physical health-related quality of life, self-efficacy, optimism and social support, and SWB, path analyses were conducted using AMOS v22 [77]. Path analysis is a powerful method of simultaneously modeling the causal relations among different variables, including one or more dependent variables and one or more independent variables, as well as one or more mediating variables. The potential direct and indirect effects of the psychosocial variables (as independent variables) on happiness (as dependent variable) with HB and LS as intermediary (mediating) variables were tested. To control for confounding variables, age, sex/gender, and institutionalization status were included in the analyses (with paths to the mediators and outcome) as continuous or binary observed variables, respectively, treated as fixed exogenous variables in a single-group model. The hypothesized model is displayed in Figure 2. The model was re-specified according to the significance of the coefficients and the modified model was retested to identify the model’s fit. The final tested model is displayed in Figure 3.

Studies have shown that the cognitive (LS) and affective (HB) elements of SWB tend to be correlated with absolute values ranging from 0.25 to 0.50, sharing a maximum common variance of 50% [78,79]. Thus, given that half as much of the variance in each dimension is explained by the other dimension, large proportions of unexplained variance remain. Thus, it is valuable to consider each dimension as a separate outcome variable rather than considering the dimensions together [26]. Furthermore, pleasant emotions have been shown to be the best predictor of happiness and LS, when other variables such as physical pleasure, satisfaction with specific domains of life, and the attainment of goals are controlled [80]. In addition, Busseri and colleagues [81,82] recently examined the tripartite structure of Diener’s proposal of SWB and have found that cross-sectional research evidence supports a causal path from happiness predictors to LS that is mediated by positive and negative affect. As a consequence, in the present study, multidimensional constructs of SWB were explored in older adults, whether predictors of happiness are related to SWB through its dimensional components was determined, and the relationships between affective and cognitive components in predicting happiness were investigated.

Based on the research by Busseri and colleagues [56,81,82], the same analyses were then conducted by first imposing a path from HB to LS and then imposing a path from LS to HB. These complementary analyses were conducted to test whether HB and/or LS can act as a mediator variable for each other in the relationship between the studied psychosocial predictors and happiness. Furthermore, they allowed us to validate previous findings on the tripartite hierarchical vs. causal structure of SWB in a study population of older adults.

As a case of structural equation modeling (SEM), the assumptions for path analysis are those required for SEM, and were satisfied by the data, as indicated previously. Maximum likelihood (ML) estimation was used as the discrepancy function for estimating standardized path effects (i.e., standardized regression coefficients, beta). Bootstrapping with 1000 samples and 95% bias-corrected confidence intervals (CI) were performed to further test the indirect effects. The goodness of fit between the theoretical model and the data-generated model was assessed by using two absolute fit indices, the chi-square statistic (χ2) and χ2/df ratio, which indicates a satisfactory fit when <3, and the standardized root mean square residual (SRMR), which indicates satisfactory fit when <0.08, as well as other incremental indices largely independent of the sample size: the noncentrality based comparative fit index (CFI) and root mean square error of approximation (RMSEA); for the CFI, the reference value is 0.95 for a satisfactory fit and 0.90 for an acceptable model, and for the RMSEA, outcomes close to or less than 0.05 indicate a satisfactory fit, and those close to or less than 0.08 indicate a reasonable fit [83]. 

Analyses were carried out without missing data. The level of significance was set to be *p* < 0.05 for all analyses.

## 3. Results

A total of 154 older individuals (50% women) took part in the study. Their age ranged from 65 to 96 years old (M = 77.44, SD = 8.03; women’s age range: 65 to 96 years, M = 78.19, SD = 7.17; men’s age range: 65 to 91 years, M = 76.67, SD = 8.79; *t* = −1.166, *p* = 0.246). Of the participants, 35.7% were institutionalized in nursing or geriatric homes with partial or complete regimens, whereas 64.3% lived in their own home (with or without relatives or other individuals). None of the participants, including those who were institutionalized, were indigent. Other sociodemographic data for the participants are presented in Table 1.

Table 2 shows the descriptive findings for all the study variables. The obtained results indicate moderate levels of psychosocial resources among the participants and poor self-reported quality of life. The participants also reported having moderate levels of SWB. Table 2 also summarizes the bivariate, zero-order Pearson’s correlation coefficients between all the study variables. The psychosocial variables correlated moderately to robustly with the SWB indicators in the expected manner, except in the case of optimism.

On the basis of insights from the correlation analysis, theoretical elaborations, and empirical findings from previous research, we hypothesized a model including the following relations. We reasoned that the influence of psychosocial resources on happiness is affected by the individual’s HB and LS. Thus, we proposed a path from perceived physical and mental health indicators, self-efficacy, optimism, and social support to HB and LS and from both components of SWB to current happiness. However, it may be that psychosocial resources are also direct predictors of happiness; thus, we included straight paths from the predictors to happiness. Institutionalization, age, and sex/gender were included as confounders. Initial analyses indicated poor model fit, and modification indices indicated that paths from self-efficacy to all other predictors should be included in subsequent analyses, as well as a path connecting social support and perceived mental health. Moreover, the findings indicated that the direct paths from the predictors to the outcome happiness were all nonsignificant and were excluded from subsequent analyses. In addition, a correlation between the mediator errors was imposed. Furthermore, all the paths from the three confounders to the mediators or the outcome were nonsignificant and were also removed. Figure 3 displays the final model as well as all the parameter estimates (standardized solution) (see also Table 3).

The model was overidentified (df > 0), and thus, the model fit was evaluated. The fit of the hypothesized model to the data was excellent, with χ2(10) = 11.837, *p* = 0.296, χ2/df = 1.184; SRMR = 0.050, CFI = 0.994 and RMSEA = 0.035.

Given that all the direct effects were nonsignificant, the psychosocial predictors influenced happiness fully through the mediators HB and LS. Physical and mental health status and social support contributed to both HB and LS and thus to happiness. Self-efficacy predicted HB but not LS, so its contribution to happiness was fully mediated by HB. Optimism predicted LS (for HB, *p* < 0.10), so its contribution to SWB was mainly due to its effects on LS. As shown in Table 4, all of the indirect effects were significant and reflected full mediation effects, except in the case of self-efficacy, for which no global indirect effect was found. The proven model explained 60%, 24%, and 28% of the variance in HB, LS, and happiness, respectively.

In addition, we conducted the same analyses by imposing a direct relationship (1) from HB to LS and (2) from LS to HB (not shown). The final redefined models had the same, optimal level of fit to the data, and similar parameter estimations were obtained. When HB was included as a predictor of LS, a regression beta of 0.29 (*p* = 0.000) was observed, and the weights of the standardized regression parameters for the remaining psychosocial predictors decreased slightly, which is consistent with the mediational role of HB [81], but no differences were observed in the pattern of the results. When LS was included as a predictor of HB, an attenuated regression beta of 0.15 (*p* = 0.000) was observed, and the same results that were reported above were observed.

## 4. Discussion

The main aim of this study was to create an explanatory model of the relationships among health-related quality of life, self-efficacy, social support, optimism, and several indicators of SWB, as well as to specify how these psychosocial resources affect HB, LS, and happiness in a study population of women and men aged 65 years or older with different personal and demographic characteristics. To that end, we investigated whether HB and LS positively correlated with psychosocial resources for happiness and whether they were mediators between these resources and SWB. Moreover, we investigated the influence of several demographic variables, with a particular focus on institutionalization, sex/gender, and age.

The expected associations among the psychosocial factors, and among HB, LS, and SWB, were confirmed. Furthermore, HB, LS, and SWB were found to be significantly correlated with the studied psychosocial resources; optimism was a notable exception in almost all these effects. In our study, while perceived physical and mental health status tended to demonstrate stronger associations with negative affect and LS, self-efficacy and social support were more strongly linked to positive affect. In addition, both the affective (i.e., HB) and cognitive (i.e., LS) indicators of SWB demonstrated strong associations with current happiness. These results confirm the theoretical proposal that affective balance and satisfaction with life are important, related, and partially independent components of SWB [10,56]; thus, the core aims of interventions designed to improve older individuals’ happiness should be focused on improving HB and LS.

Moreover, we reasoned that the effects of psychosocial resources on happiness have an influence on, and are influenced by, the individual’s HB and LS states. Thus, we proposed a path model from perceived physical and mental health indicators, self-efficacy, optimism, and social support (predictors) to HB and LS (mediators) and from the two latter factors to current happiness (outcome). All of the indirect effects were significant and indicated full mediation, except in the case of self-efficacy. Among older adults, SWB increases by approximately 0.3 units for every 1-unit increase in HB and LS, and HB and LS increase up to 0.4 units and 0.3 units, respectively, for every 1-unit increase in the predictor psychosocial resources. As the indirect effects revealed, the findings demonstrate that physical health, social support, and especially mental health are important for the elderly people’s well-being [30]. A total of 28% of the variance of the outcome happiness was explained; however, there is still a notable proportion of unexplained variance. Additional research should be conducted to assess other predictors of SWB in older adults.

Our findings are in line with those supporting a positive relationship between social support [27,28,32,33] and health status [27,31,33,43,45] and HB and LS. Furthermore, our findings are contradictory for optimism. While no significant pairwise correlations with HB, LS, and happiness were found, optimism predicted LS in our model [44,45,52]. Consistent with this unexpected finding for optimistic expectancies, a previous study showed that an intervention focused on increasing optimism did not reveal any effect on SWB among Spanish older adults [84]. Consequently, future research on the role of optimism for SWB in late adulthood is warranted. It would also be of interest to explore whether optimism might in turn be mediated by or mediate—as our findings seem to indicate—the effects of other variables, such as self-efficacy, with which it showed a unique significant correlation [52]. Notably, none of the reviewed studies analyzed whether HB and LS are mediating variables for SWB when these predictors are considered, which is a contribution of the present study.

However, contrary to other findings [26,44,50,53], the findings in our study did not suggest any relationships of self-efficacy with SWB. Self-efficacy predicted HB but was not correlated with LS, so its global indirect effect was nonsignificant. Note that a modification was introduced to the first model by imposing a direct path from self-efficacy to the remaining predictors. Consequently, we believe that self-perceptions of efficacy have a predictive effect on the remaining psychosocial resources; if self-efficacy does not act on social support, optimism and physical and mental health, these factors would have no effect or would have a smaller effect on the mediating variables and thus on happiness. Future research must be conducted to more deeply explore the effects of self-efficacy on HB, LS, and overall SWB. Moreover, a negative value for the weight of the association between self-efficacy and LS was found in the path analysis, contrarily to pair-wise correlations, indicating inconsistent mediation [85] and probably a suppression effect by which other variables (e.g., coping) might be moderating the influences of personal efficacy on happiness among elderly people. More research is needed to explore this possibility further.

Institutionalization, age, and sex/gender were included as confounding factors. None of the three sociodemographic variables demonstrated a relevant contribution, which is inconsistent with other findings [27,49] and consistent with some other findings [56,86], so our results show the generalizability of the proposed model. Moreover, our findings support those of other authors who argued that psychosocial resources are the most important correlates of HB (positive and negative affect) and LS [27,33,43]. Fortunately, psychosocial resources are modifiable, in contrast to many sociodemographic variables. Identifying the resources with the highest impact on HB and LS can help in enhancing SWB, as these resources can be changed through appropriate interventions. However, some authors have found that institutionalization may moderate the relationships between psychosocial resources for well-being and SWB in older adults [29,32]. We did not observe this result, and future research is needed to explore the influence of institutionalization on older individuals’ happiness.

Finally, the present study contributes relevant evidence on the structure of SWB and the relationships among its components in the elderly population. In a series of studies analyzing the tripartite structure of SWB with young and adult study populations, Busseri and colleagues [56,81,82] provided empirical evaluations of several competing structural approaches regarding the relationships among HB, LS and SWB. In a meta-analytic study with more than 34,000 individuals and cross-sectional, longitudinal and experimental data, Busseri [56] confirmed that SWB has a hierarchical structure and found moderate meta-analytic correlations among the three proposed components (−0.49 for positive affect and negative affect, 0.53 for positive affect and LS and −0.37 for negative affect and LS), stronger correlations between these factors, and SWB (.84 for positive affect, −0.54 for negative affect and 0.63 for LS; the factor SWB explained 70%, 34% and 40% of their variance, respectively) and reliable unique variance for each component. This model demonstrated robustness and generalizability, as several moderators, including age, sex, study population composition, and cultural background, showed a lack of significant effects. Consequently, Busseri [56] claimed that a hierarchical conceptualization of SWB is “the only structural model that is consistently supported, and not contradicted, by the available empirical evidence” (p. 70); thus, both the shared and unique variances of HB and LS are critical for understanding SWB. Accordingly, global life evaluations and affective experiences may have similar as well as unique characteristics in terms of associations with other variables of interest, potential causes and consequences, and stability/change over time.

However, there is some evidence [81] supporting an alternative structure in which, within a causal system, HB or its components unidirectionally influence LS and act as potential mediators of the influence of other variables (e.g., psychosocial resources) on LS, which has been typically treated as the SWB outcome in the literature. Nevertheless, this proposal was confirmed only when cross-sectional data rather than longitudinal data were analyzed, which provided some evidence for a reverse pathway. The author of the study also claimed that the mediating effects of HB on the path between predictors and LS need to be better understood. In subsequent studies [82], it was confirmed that while cross-sectional data supported this structure, longitudinal and experimental data did not; moreover, evidence was also found of a bidirectional relationship between affective components of SWB and LS.

As a consequence, researchers were encouraged to further investigate the correlates, predictors and consequences of SWB based on a hierarchical tripartite structure [82]. The present study responds to this claim, as we have explored the predictive role of several psychosocial resources by discriminating their unique contributions to happiness and its individual components, HB and LS. The results support the hierarchical structure [56] and do not support a causal system model by which HB contributes unidirectionally to LS and acts as a mediator. Thus, our study contributes to the knowledge on the sources of happiness, their unique contributions, and psychological consequences in older adults.

It is worth noting that we mixed mood in a unique indicator of HB. Other authors have found diverging results for positive and negative affect. It has been found that the association between positive affect and LS is stronger than that between negative affect and LS [26]. Similarly, among older adults it has been found that both positive and negative affect predicted LS; however, positive affect was almost four times more likely to predict satisfaction with life than was negative affect [86]. The authors concluded that interventions seeking to improve overall well-being should focus on increasing positive affect rather than decreasing negative affect.

As life expectancy is rising worldwide, it is becoming increasingly important to identify the factors that can contribute to more successful and joyful aging. The size of the global population of adults aged 60 or more surpassed 900 million in 2017 (12%) and is expected to double by 2050, when it is projected to reach nearly 2.1 billion (22%) [87,88]. This increase is likely to result in considerable health, economic and societal burdens, and has implications for individuals’ well-being, a key component of successful aging [44]. Thus, the need for interventions aimed at increasing older individuals’ resources for enhanced quality of life and overall well-being, positive and healthy aging, and a reduction of risks for disease and disability will be increasingly more needed. Despite the study limitations, the present findings contribute to the existing literature on happiness in the elderly population. Our results are novel and interesting; they highlight the importance of psychosocial variables to be considered in order to improve emotional well-being, satisfaction with life and thus happiness in older adults. These factors should be promoted by interventions, which should also inform older adults about social and community resources available that may enhance psychosocial correlates of SWB.

This study has limitations that should be noted. The main limitation is that the sample size is small, and studies with larger sample sizes on this same topic need to be conducted. Furthermore, as the participants were volunteers with an adequate health status and level of cognitive functioning and overrepresented community-dwelling older adults with low-to-moderate education and socioeconomic levels, they might not be representative of older adults in Spain or other countries. Our study population appeared, however, roughly comparative to those included in national databases and other studies conducted in Spain, particularly in southern Spain. Second, our findings completely rely on self-reported data; the inclusion of other sources of information (e.g., informant reports, clinical reports, behavioral observations, experience-based sampling) would increase the validity of our results. Moreover, a revision of some measures (e.g., dispositional optimism [89]) might lead to greater consistency in the empirical findings. Furthermore, we explored only some variables related to SWB, while many other variables were ignored (e.g., stress and coping, personality traits, motivational styles). Future research should include more of the well-established factors that have been linked to SWB. Third, the correlational, cross-sectional design of this study does not allow us to make causal conclusions. A longitudinal, prospective study examining changes in the relationships among the variables over time or an experimental study including an intervention aimed at increasing psychosocial resources for enhanced SWB may provide more exact interpretations of the relationships among self-efficacy, optimist expectations, social relationships, and well-being outcomes and may also allow the predictive validity of the model to be tested or possible differential SWB patterns that occur over time to be explored. Finally, cultural differences have been identified in both optimist and pessimist expectancies for the future [90], self-efficacy [91], and social support [92]. Diener et al. [93] also stressed that cultural variables explain differences in mean levels of SWB both at the individual and community levels, and culture has been frequently mentioned when explaining national differences in SWB [94,95]. Thus, the results presented herein should be verified in studies including individuals from other nations and individuals with different ethnic and cultural backgrounds.

## 5. Conclusions

In summary, increasing one’s personal skills and perceptions of personal competence, the provision of more social support and social leisure opportunities, increasing one’s optimistic expectancies, and promoting healthy lifestyles as well as good physical and mental health and daily functioning may increase individuals’ levels of HB and LS and consequently help older adults feel greater levels of SWB. Our results point to one fundamental idea: while perceived physical and mental health, social support, and optimism are not only associated with better emotional well-being and higher psychological satisfaction but also have important implications for overall SWB, self-efficacy plays a fundamental role as a psychosocial resource that empowers other individual resources, thereby increasing the impact of psychosocial resources on HB and LS and thus happiness.

## Figures and Tables

**Table 1:** Socio-demographic characteristics of the participants.

Condition		%
Marital Status	Single with no history of a regular partner	1.9
	Married or in a stable relationship	56.5
	No current partner but a history of a stable or marital relationship	41.6
Education Level	No education	20.8
	Primary	46.1
	Secondary	16.9
	Vocational training	7.1
	University	9.1
Employment Status	Working	1.9
	Unemployed	2.6
	Housework	20.8
	Retired	74.7
Monthly Family Income	<1000 €	47.6
	1000–2000 €	46.2
	2000–3000 €	4.2
	>3000 €	2.1
Number of Children	0	10.4
	1	3.2
	2	23.4
	3	31.8
	≥4	31.1
Chronic Disease (Perceived health on a 0–10 points scale: M = 6.25, SD = 1.89)	Yes, of which (more frequent)	74.7
	Cardiovascular diseases including coronary and vascular diseases, hypertension and high cholesterol	47.9
	Bone and rheumatic diseases including osteoporosis, osteoarthritis, and rheumatoid arthritis	27.1
	Diabetes	6.9
	Hyperthyroidism	4.2
	Neurologic diseases including multiple sclerosis, Parkinson, and limb paralysis	3.5
	Respiratory diseases including asthma and chronic bronchitis	2.8
	Limb amputation	2.8
	Cancer, including prostate diseases	2.8
	Sensorial deficit	2.1
Any Kind of Therapy	Yes, of which (more frequent)	76.6
	Cardiovascular treatments	26.5
	Non-steroidal anti-inflammatory drugs	19.7
	Calcium and vitamins supplements	6.8
	Diabetes treatments	5.1
	Hyperthyroidism treatments	4.3
	Anxiolytics	3.4

Note. A third of the participants reported taking some kind of drugs but were unable to specify their correct active principles, names, or aims, and it was not possible to know this information based on clinical status or records.

**Table 2:** Descriptive findings and zero-order *r* correlations for all the study variables (*N* = 154).

	Min	Max	*M*	*SD*	1	2	3	4	5	6	7	8	9	10
1. SF12PCS	16.66	60.30	39.08	10.86	-	0.11	0.43 **	0.06	0.18 *	0.35 **	−0.46 **	0.48 **	0.24 **	0.29 **
2. SF12MCS	20.73	64.11	46.59	9.93		-	0.26 **	0.05	0.14 †	0.36 ^**^	−0.58 **	0.55 **	0.33 **	0.36 **
3. Self-efficacy	46	99	78.67	11.32			-	0.17 *	0.29 **	0.44 ^**^	−0.39 **	0.49 **	0.17 *	0.23 **
4. Optimism	10	29	19.83	2.76				-	–0.10	0.13	−0.03	0.10	0.13	0.01
5. Social support	37	95	72.56	16.81					-	0.48 **	−0.34 **	0.49 **	0.32 **	0.34 **
6. Positive affect	9	27	16.87	3.92						-	−0.43 **	0.85 **	0.29 **	0.40 **
7. Negative affect	9	26	15.88	3.80							-	−0.84 ^**^	−0.51 **	−0.40 **
8. Hedonic balance	−13	17	0.99	6.53								-	0.47 **	0.47 **
9. Life satisfaction	8	35	25.88	5.61									-	0.45 **
10. Current happiness	0	10	6.59	1.90										-

SF12PCS: SF12 Physical health component summary; SF12MCS: SF12 Mental health component summary. † *p* < 0.10, * *p* < 0.05, ** *p* < 0.01.

**Table 3:** Standardized regression weights.

	Path		Stand. Estimate	S.E.	C.R.	*p*
SF12PCS	-	Hedonic balance	0.32	0.040	2.176	0.000 **
SF12MCS	-	Hedonic balance	0.46	0.042	4.296	0.000 **
Self-efficacy	-	Hedonic balance	0.13	0.042	−1.107	0.025 *
Optimism	-	Hedonic balance	0.10	0.145	2.378	0.068 †
Social support	-	Hedonic balance	0.33	0.025	3.792	0.000 **
SF12PCS	-	Life satisfaction	0.17	0.033	5.758	0.030 *
SF12MCS	-	Life satisfaction	0.32	0.034	8.643	0.000 **
Self-efficacy	-	Life satisfaction	−0.09	0.034	2.240	0.268
Optimism	-	Life satisfaction	0.17	0.119	1.822	0.017 *
Social support	-	Life satisfaction	0.28	0.020	6.238	0.000 **
Hedonic balance	-	Happiness	0.33	0.023	4.220	0.000 **
Life satisfaction	-	Happiness	0.29	0.026	3.815	0.000 **
Self-efficacy	-	SF12PCS	0.39	9.791	4.716	0.000 **
Self-efficacy	-	SF12MCS	0.24	8.334	3.130	0.002 **
Self-efficacy	-	Optimism	0.16	2.174	2.289	0.022 *
Self-efficacy	-	Social support	0.26	14.185	3.384	0.000 **
Social support	-	SF12MCS	0.19	13.651	2.305	0.021 *
e1	-	e2	0.21	1.605	2.514	0.012 *

SF12PCS: SF12 Physical health component summary; SF12MCS: SF12 Mental health component summary. e1: error term for Hedonic Balance. e2: error term for Life Satisfaction. † *p* < 0.10, * *p* < 0.05, ** *p <* 0.01.

**Table 4:** Standardized indirect effects (outcome: happiness).

Predictor	Stand. Estimate	95% CI Lower Bound	95% CI Upper Bound	*p*
SF12PCS	0.16	0.088	0.229	0.004 **
SF12MCS	0.24	0.157	0.326	0.002 **
Self-efficacy	0.02	−0.079	0.080	0.770
Optimism	0.08	0.024	0.153	0.007 **
Social support	0.19	0.109	0.269	0.003 **

SF12PCS: SF12 Physical health component summary; SF12MCS: SF12 Mental health component summary. ** *p* < 0.01.
